# Associations between Practitioner Personality and Client Quit Rates in Smoking Cessation Behavioural Support Interventions

**DOI:** 10.1017/jsc.2017.10

**Published:** 2018-06

**Authors:** Heather L. Gainforth, Sarita Y. Aujla, Emma Beard, Emma Croghan, Robert West

**Affiliations:** 1 School of Health and Exercise Sciences, University of British Columbia, Kelowna, Canada; 2 Department of Epidemiology & Public Health, University College London, London, UK; 3 North51, Nottingham, UK; 4 The Department of Behavioural Science and Health, University College London, London, UK

## Abstract

**Introduction:** There is wide variation in the success rates of practitioners employed to help smokers to stop, even once a range of potential confounding factors has been taken into account.

**Aim:** This paper examined whether personality characteristics of practitioners might play a role success rates.

**Methods:** Data from 1,958 stop-smoking treatment episodes in two stop-smoking services (SSS) involving 19 stop-smoking practitioners were used in the analysis. The outcome measure was clients’ biochemically verified quit status 4 weeks after the target quit date. The five dimensions of personality, as assessed by the Ten-Item Personality Inventory, were included as predictor variables: openness, conscientiousness, agreeableness, extraversion, and neuroticism. A range of client and other practitioner characteristics were used as covariates. A sensitivity analysis was conducted to determine if managers' ratings of practitioner personality were also associated with clients’ quit status.

**Results:** Multi-level random intercept models indicated that clients of practitioners with a higher extraversion score had greater odds of being abstinent at four weeks (self-assessed: OR = 1.10, 95% CI = 1.01–1.19; manager-assessed: OR = 1.32, 95% CI = 1.21–1.44).

**Conclusions:** More extraverted stop smoking practitioners appear to have greater success in advising their clients to quit smoking. Findings need to be confirmed in larger practitioner populations, other SSS, and in different smoking cessation contexts. If confirmed, specific training may be needed to assist more introverted stop smoking practitioners.

## Introduction

Research has demonstrated that behavioural and pharmacological support for smoking cessation increases abstinence rates (NICE, 2008; West, 2012; West & Brown, 2012; West, McNeill, & Raw, 2000). In the United Kingdom, the English stop-Smoking Services (SSS) offer free, evidence-based behavioural and pharmacological support to those motivated to quit (Department of Health, 1998). It is estimated that smokers are approximately four times more likely to succeed in quitting using the specialist support of this kind plus medication than if they quit unaided (West, 2012; West & Brown, 2012).

Success rates of individual services vary widely (National Health Service Information Centre, 2011). Individual practitioners also differ in success rates. An examination of 4-week biochemically verified quit rates from 31 SSS in England found that after confounding factors such as age, gender, and nicotine dependence were controlled for, practitioners still accounted for 7.6% of the variance in success rates, indicating that different practitioners differ in their effectiveness (Brose, McEwen, & West, 2012). It is important to understand how differences in practitioner factors may be associated with the variation in client outcomes.

Practitioner personality may explain some of the variations in success rates among stop smoking practitioners. Practitioner personality may influence the quality of the relationship with clients in a way that affects outcomes. Also, practitioner conscientiousness may influence how thoroughly and carefully the practitioner applies the evidence-based behaviour change techniques that form the core of the behavioural support interaction (Michie et al., 2013; West, Walia, Hyder, Shahab, & Michie, 2010).

For the purpose of the present study, personality was defined as a consistent and enduring way of thinking, feeling, and behaving that characterises an individual (Carver & Scheier, 2000) and was measured according to a five-factor model (FFM) of personality (Costa & McCrae, 1992). The FFM is a dominant model of personality and hierarchically organises the personality traits into five dimensions: openness-to-experience (a measure of intelligibility and creativity); conscientiousness (a measure of pro-activity and discipline); extraversion (a measure of sociability and warmth); agreeableness (a measure of friendliness and altruism); and neuroticism (a measure of anxiety and other negative emotions) (Costa & McCrae, 1992). While other models of personality may be relevant, the FFM provides a good starting point because of its reliability and validity both within and across cultures (McCrae, 2002).

Research in other areas of counselling has found that practitioner personality may play a role in outcomes. While measures of key variables are heterogeneous in the literature, studies have found a positive association between practitioner agreeableness and client outcome (Engvik, 1999; Lafferty, Beutler, & Crago, 1989; Marlett, 2008; Miller, Taylor, & West, 1980; Najavits & Strupp, 1994; Valle, 1981; Williams & Chambless, 1991). Support for an association between the other personality dimensions is less robust. A small number of studies have found negative associations between practitioner neuroticism and client outcomes, positive associations between conscientiousness and client outcomes, and varied associations between client outcomes and practitioner extraversion (Engvik, 1999; Rosenberg, Gerrein, Manohar, & Liftik, 1976; Snowden & Cotler, 1974).

The primary aim of the present study was to determine if stop smoking practitioners’ self-assessed FFM personality scores were associated with clients’ 4-week biochemically verified quit status. The secondary aim was to conduct a sensitivity and exploratory analysis to determine whether SSS managers' observer-reported FFM ratings of practitioner personality were associated with clients’ 4-week biochemically verified quit status. Findings will provide preliminary indication as to whether managerial ratings could be used to assess practitioner personality and ultimately success. Given previous findings, it was hypothesized that the FFM dimension of agreeableness would be associated with greater odds of clients being biochemically verified as abstinent at 4 weeks (Williams & Chambless, 1991). Given the limited literature, hypotheses for the other four FFM dimensions were not formulated.

## Methods

### Design

This was a correlational study with each of five practitioner personality dimensions used to predict abstinence in clients, adjusting for potential confounding variables. Data were sourced from two English SSS using QuitManager, an online database system that adheres with the UK Department of Health's monitoring guidance for local SSS (Croghan, 2011). All data were anonymised and judged by the University College London Ethics Committee to not to require approval.

### Participants

Data were collected from *three* groups of participants: clients, practitioners, and managers. Outcome data were collected from clients and personality scores were collected from *both* practitioners and managers.

Clients were included if: they had completed a treatment episode between April 1 2013 and April 1 2014 and were recorded as aged 12–90 years. A treatment episode was defined as a one quit attempt as characterised by follow-up with a stop smoking practitioner to set a quit date (Croghan, 2011). North51 offers clients private, one-to-one consultations with a practitioner. The consultations can take place face-to-face in a clinic or community setting like a village hall, GP practice, or over the phone. Clients are offered weekly appointments for the first few weeks and then either continue with the weekly appointments, or move to fortnightly appointments until abstinence is reached at 3 months.

The practitioners (*n* = 19) were all employed by the two participating services to give one-to-one behavioural support to smokers wanting to stop. All practitioners had completed training from the National Centre for Smoking Cessation and Training (NCSCT; for training details see www.ncsct.co.uk/) and were asked to follow the same treatment manual.

The managers (*n* = 2) were employees of the participating SSS. Both managers directly oversaw the training and delivery of services provided by the practitioners included in the sample.

### Measures

For each client, measures of age, sex, ethnicity (white/non-white), smoking cessation medication use (yes/no), and social grade (low/medium/high) were extracted from QuitManager. For each practitioner, measures of age, sex, and experience as a stop smoking practitioner (in years) were obtained from QuitManager.

The Ten-Item Personality Inventory (TIPI) (Gosling, Rentfrow, & Swann, 2003) was used to measure the FFM dimensions of personality (see http://gosling.psy.utexas.edu/scales-weve-developed/ten-item-personality-measure-tipi/). The TIPI consists of two items per FFM dimension, each measured on a seven-point scale. The TIPI takes approximately 5 min to complete (John & Srivastava, 1999). A comparison of the TIPI and the larger Big Five Inventory (BFI) in terms of convergent validity, discriminant validity, and test–retest reliability has indicated that the TIPI is a psychometrically satisfactory proxy for the longer instrument (Gosling et al., 2003).

In the present study, the TIPI was completed by 19 practitioners and 2 managers (i.e., as a self-assessment questionnaire by practitioners and as observer-reported questionnaire by managers). Practitioners were directed to ‘write a number next to each statement to indicate the extent to which you agree or disagree with that statement’ (1 = *disagree strongly*, 7 = *agree strongly*) for 10 statements starting with ‘I see myself as’. Managers were directed to ‘write a number next to each statement to indicate the extent to which you agree or disagree with that statement about the Stop Smoking Practitioner’ for the identical 10 statements but instead beginning with ‘I see the practitioner as’. To our knowledge, the TIPI has previously not been used as an observer-reported questionnaire. Therefore, manager ratings were only used to conduct sensitivity and exploratory analyses.

Consistent with the UK Department of Health's monitoring guidance (Croghan, 2011), a successful quit attempt by clients was defined as biochemically verified smoking abstinence, measured at 4 weeks following the target quit date. Biochemical verification involved an expired-air carbon monoxide concentration <10 ppm. Consistent with guidance (Croghan, 2011), clients who did not attend the 4-week follow-up were presumed to be smoking.

### Analysis

Descriptive statistics and inter-rater agreement were derived using SPSS version 22. Two TIPI scores for each of the FFM personality dimensions were calculated for each practitioner in the data set: (1) practitioners’ self-assessed score; and (2) mean managers’ observer-reported score. To determine the inter-rater consistency between the two managers’ observer-reported TIPI scores and between the managers’ observer-reported ratings and practitioners’ self-assessed TIPI scores, two-way random intra-class correlation coefficients (ICCs) were calculated. ICCs < 0.40 were considered to be poor, 0.40–0.59 to be fair, 0.60–0.74 to be good, and 0.75–1.0 to be excellent (Cicchetti, 1994).

Given the clustered nature of the data, a likelihood ratio test was run to determine whether a multilevel model specifying random intercepts was required. The likelihood ratio test compared the random intercept only model with a null single-level model. A significant effect was found, *χ*^2^ = 37.99, *p*<0.001 but not the repeated measures (*χ*^2^ = 7.24, *p*>0.001), and so a model accounting for clustering was used. More conservative *p* value cut-offs are used when deciding on the inclusion of level 2 random effects due to their increased cost in terms of power and over parameterisation (Finch, Bolin, & Kelley, 2014). Sensitivity analyses showed that the direction and size of the results were unchanged when accounting for the repeated measures. Thus, models were run with level 2 random intercepts for the practitioner clustering.

The sample size recommendations for multilevel regression analysis are at least 20 clusters with more than 30 observations in each (Heck, 2001), and at least 10 observations per predictor variable (Peduzzi, Concato, Kemper, Holford, & Feinstein, 1996). The adjusted analyses included 14 independent variables, which would have required a minimum sample size of 140. However, given that the sample size for multilevel models depends on the effect size being considered and extent of intra-cluster correlation, it is recommended that researchers use simulation methods to ascertain power (Moineddin, Matheson, & Glazier, 2007). Thus simulation-based power calculations were produced assuming a baseline quit rate of 50% (Health and Social Care Information Centre, 2015), 20 clusters (i.e., 20 practitioners) with 50 participants in each cluster, and OR for detecting an association between one personality trait dimension and carbon monoxide (CO)-validated 4-week quit status of 1.2 (Hakulinen et al., 2015). Personality trait scores were assumed to occur on a 14-point scale and were randomly generated using a Poisson distribution with mean of 7. It was found that with this sample the study would have >90% power to detect a difference in quit rates if the intra-cluster correlation was <0.5%.

Two multi-level random intercept models were fitted with a random effect for the practitioners to account for clustering. Both models were run using R version 2.3.1 and the glmer() function in the lme4 package, specifying the binomial family and maximum likelihood estimation (MLE) (Bates & Sarkar, 2006). In the first model testing our primary hypothesis, client and practitioner characteristics were entered as covariates and practitioners’ self-assessed scores for each of the FFM dimensions were entered as separate predictor variables. In the second model testing our secondary hypothesis, client and practitioner characteristics were again entered as covariates. Mean manager observer-reported scores for each of the FFM dimensions were entered as separate predictor variables.

## Results

Data from treatment episodes completed by 19 stop-smoking practitioners were included in the analysis (79.0% female; *M*_age_ = 48.86 ± 10.33 years). The experience of practitioners varied considerably (*M*_years_ = 5.40 ± 3.14, range = 1.0–12 years). During the period between April 1 2013 and April 1 2014, 2,132 treatment episodes^1^ with clients were entered into QuitManager. After missing data were removed due to data entry error (*n* = 169) and inclusion criteria applied, a final sample of 1,958 treatment episodes with clients remained (54.1% female, *M*_age_ = 42.12 ± 15.86 years). Among these clients, 84.7% (*n* = 1659) received medication to aid in their quit attempt and 38.4% (*n* = 752) had quit smoking at 4 weeks. Clients were primarily white (89.5%, *n* = 1,752); 24.7% (*n* = 483) were of low social-grade, 22.7% (*n* = 445) of medium social-grade, and 52.6% (*n* = 1,030) of high social-grade.

Mean practitioner self-assessed and managers’ observer-reported TIPI scores for each of the FFM dimensions are presented in Table 1. ICCs revealed that agreement between the two managers’ observer-reported scores was excellent for the dimensions of neuroticism and agreeableness, fair for the dimension of conscientiousness, and poor for the dimensions extraversion and openness-to-experience (see Table 1). Agreement between the mean manager observer-reported scores and practitioners’ self-assessed scores was good for the dimension of extraversion and poor for the remaining four dimensions (see Table 1).

**Table 1:** Self-assessed and observer-reported TIPI scores for the FFM dimensions

Personality	Self-assessment,	Managers assessment,	ICC self-assessed vs.	ICC manager 1 vs.
dimension	mean (SD)	mean (SD)	managers, score (CI)	manager 2, score (CI)
Extraversion	9.95 (2.44)	10.45 (1.70)	0.61(−0.01–0.85)*	0.23 (−1.01–0.70)
Neuroticism	10.50 (2.39)	7.89 (2.86)	0.36 (−0.66–0.75)	0.82 (0.54–0.93)*
Agreeableness	11.26 (2.38)	9.34 (2.33)	−0.09 (−1.83–0.58)	0.77 (0.40–0.91)*
Conscientiousness	11.32 (2.11)	9.71 (2.16)	−0.39 (−2.60–0.47)	0.56 (−0.13–0.83)*
Openness	11.37 (1.86)	9.16 (1.33)	−0.41 (−2.67–0.46)	0.37 (−0.63–0.76)

*p< 0.05.

Results of the first multi-level random intercept model testing our primary hypothesis indicated that clients who were seen by a practitioner with a higher self-assessed extraversion TIPI score had greater odds of being CO-validated as abstinent from smoking at 4 weeks (OR = 1.10, 95% CI = 1.01–1.19). None of the other four self-assessed FMM dimensions were found to be associated with clients’ CO-validated 4-week quit status (see Table 2). This finding was supported by the results of our second multi-level random intercept model, which indicated that managers’ mean observer-reported extraversion score was associated with clients’ CO-validated 4-week quit status, with higher observer-reported extraversion scores being associated with greater odds of being CO-validated as abstinent at 4 weeks (OR = 1.32, 95% CI = 1.21–1.44). Mean manager observer-reported scores for the dimensions of agreeableness were positively associated with clients’ CO-validated 4-week quit status (OR = 1.21, 95% CI = 1.08–1.36), whereas conscientiousness was negatively associated with clients’ quit status (OR = 0.85, 95% CI = 0.76–0.96). Openness-to-experience and neuroticism were not found to be associated with clients’ CO-validated 4-week quit status (see Table 2).

**Table 2:** Results of the multi-level analyses assessing the association between confounding factors and practitioner personality traits with CO-validated 4-week quit status

	OR	95% CI	
*Model 1: Self-assessed personality scores*			
Client sex (female)	1.13	0.93	1.37
Client ethnicity (white)	1.06	0.76	1.49
Medication (no medication)	1.44*	1.04	2.00
Client social grade			
Medium	1	1	1
High	1.07	0.81	1.42
Low	0.92	0.72	1.16
Client age at quit date	1.02***	1.01	1.02
Practitioner experience, years	1.07	0.99	1.15
Practitioner sex (female)	1.44	0.78	2.65
Practitioner age, years	0.99	0.97	1.02
Extraversion	1.10*	1.01	1.19
Agreeableness	0.99	0.89	1.10
Conscientiousness	0.94	0.81	1.08
Neuroticism	1.03	0.95	1.12
Openness	1.07	0.93	1.22
*Model 2: Observer-reported personality scores*			
Client sex (female)	1.11	0.92	1.35
Client ethnicity (white)	0.93	0.66	1.31
Medication (no medication)	1.55**	1.14	2.10
Client social grade			
Medium	1	1	1
High	1.12	0.85	1.48
Low	0.97	0.77	1.24
Client age at quit date	1.02***	1.01	1.02
Practitioner experience, years	0.99	0.94	1.03
Practitioner sex (female)	1.80*	1.12	2.91
Practitioner age, years	1.01	1.00	1.03
Extraversion	1.32***	1.21	1.44
Agreeableness	1.21**	1.08	1.36
Conscientiousness	0.85**	0.76	0.96
Neuroticism	1.03	0.91	1.17
Openness	0.93	0.78	1.11

*Note:* Reference categories are denoted in parentheses. ***p<0.001; **p<0.01; *p<0.05.

## Discussion

Stop-smoking practitioners with higher extraversion scores had higher success rates than those with lower scores. This finding builds on the previous, although limited, counselling research examining the association between practitioner personality and client outcomes.

Extraversion is characterised by liveliness, enthusiasm, and sociability, and has been found to be a predictor of general confidence and social interaction (Cheng & Furnham, 2002). A possible explanation for the association between extraversion and success rates is that more extraverted practitioners may be more confident or find it easier to engage with the client during behavioural support interactions. Introverted practitioners may also be less talkative and outgoing during interactions with clients. Evidence from psychotherapy suggests that quiet and reserved therapists produce lower levels of client satisfaction (Conte, Buckley, Picard, & Karasu, 1994). Extraverts’ confidence and talkativeness may also allow them to apply evidence-based behaviour change techniques more easily and confidently (Michie et al., 2013; West et al., 2010). To understand the mechanisms that may explain our findings, experimental research examining practitioner personality and the practitioner–client interaction in smoking cessation behavioural support interventions is needed.

The failure to find a clear association between other personality dimensions and client success rates may be because the other dimensions are less relevant or may relate to poor measurement. It is noteworthy that associations between managers’ ratings of practitioner agreeableness and conscientiousness were found to be associated with clients’ being abstinent from smoking. Given that validity and reliability of observer-reported TIPI ratings have not been assessed and due to the poor consistency between managers’ scores and practitioners’ scores, the validity of these associations with quit status must be questioned. Managers play a key role in the SSS; therefore, understanding how managers can reliably and validly assess practitioner personality demands further research.

If practitioner extraversion is associated with higher success rates, training programmes that target the manner in which practitioners interact with clients may be useful. While practitioners’ personalities will not be altered, it may be possible to change their interactional style to one that is more engaging. However, this will require study as to what aspects of the interaction mediate the association between personality and client outcomes.

The study had several limitations. First, a brief measure of personality was used. It may be possible to obtain more accurate measurement of personality with the more detailed, yet still concise, BFI personality measure (Benet-Martinez & John, 1998). A different model of personality may also result in different dimensions emerging as relevant. Second, the study was limited to 19 practitioners working in two services. It will be important to establish how far these findings extend in a larger sample. Third, as a correlational study, caution must be exercised in drawing causal conclusions. It is difficult to conceive of ways in which the causal association could be reversed or how other factors might confound the association given that smokers do not choose the practitioners with whom they consult. However, further research should consider a wider range of potential confounding variables such as practitioner training, therapeutic alliance between the client and practitioner, and client personality. Fourth, the study only assessed short-term outcomes. It is possible that longer term cessation is less influenced by factors involved in the client–practitioner interaction. Fifth, clients who were lost to follow-up were presumed to be smoking. This approach is consistent UK Department of Health's monitoring guidance for the SSS (Croghan, 2011). However, it is possible that practitioner personality may affect clients’ willingness to return to the service rather than their smoking status.

## Conclusions

Overall, the findings provide further understanding as to how practitioner personality may affect success rates in the SSS. Extraverted stop-smoking practitioners appear to have greater success in advising their clients to quit smoking. If this finding is confirmed, it could indicate a useful area of focus for training practitioners.

## Endnote

1 85% (*n* = 1,160) of participants only accessed the services one. The remaining 15% of participants (*n* = 298) accessed the services more than twice.

## Appendix

**Abbreviations**
SSS: Stop-smoking services
FFM: Five-factor model
NCSCT: National Centre for Smoking Cessation and Training
TIPI: Ten-Item Personality Inventory
ICC: Intra-class correlation
MLE: Maximum likelihood estimation
CO: Carbon monoxide
